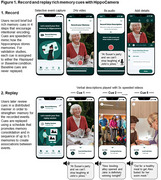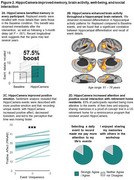# An easy‐to‐use smartphone intervention that improves memory for real‐world recent events

**DOI:** 10.1002/alz70858_099512

**Published:** 2025-12-24

**Authors:** Morgan D Barense, Bryan Hong

**Affiliations:** ^1^ University of Toronto, Toronto, ON, Canada

## Abstract

Memory is essential for shaping how we interpret the world, plan for the future, and understand ourselves, yet effective cognitive interventions for real‐world episodic memory loss remain scarce. This talk introduces HippoCamera, a smartphone‐based behavioural intervention inspired by how the brain supports memory, designed to enhance real‐world episodic recollection by replaying high‐fidelity autobiographical cues. We will present new work from a HippoCamera intervention in a residential living facility, as well as a HippoCamera intervention delivered entirely remotely. The perspective will highlight how this behavioural intervention can improve memory and mood, while uncovering links between memory distinctiveness, well‐being, and the perception of time.